# The management of skeletal dysplasia

**DOI:** 10.1186/1687-9856-2013-S1-O19

**Published:** 2013-10-03

**Authors:** Tae-Joon Cho

**Affiliations:** 1 Seoul National University Children’s Hospital, Seoul, Korea

## 

Skeletal dysplasia (SD) is disorders of bone and cartilage development caused by genetic defects. It comprises more than 300 specific disorders[1]. Causative genes responsible for SD include those encoding the bone and cartilage matrix proteins, receptors and intracellular molecules involved in signal pathway of chondrocyte and osteoblast, specific transcription factors, enzymes involved in bone and cartilage metabolism, cell surface ion channels, and so on. Recently, many novel causative genes have been discovered with the help of whole exome sequencing, whose protein function has not been well elucidated. SD had been classified according to their clinical and radiologic phenotype, however with discovery of causative genes, nosology and classification has evolved over the last several decades based more on the genotype. Correct diagnosis is the starting point of optimal management of SD, but it can be very difficult in some cases. One should not hesitate to consult to SD specialists in order to make correct diagnosis. Common clinical problems in the skeletal system that interfere with patients’ daily life include short stature, limb deformity, spine deformity, C1-2 instability, and precocious osteoarthritis. Only few medical treatments effective for SD have been established. Bisphosphonate for osteogenesis imperfecta was the most successful medical treatment so far. Hence, the management of SD is still largely surgical intervention and rehabilitation. Orthopedic correction of the limb deformity can improve not only cosmesis, but also patients’ locomotive function. Distraction osteogenesis by Ilizarov principle makes complex deformity correction possible, and asymmetrical physeal suppression enables minimally invasive correction of the deformity. Bilateral limb lengthening for height became popular since 80’s, but considering its high rate of complication, surgical indication should be narrowed and the surgery should be discussed thoroughly with the patient and parents preoperatively. Many SDs provoke hip problems and various surgical interventions have been applied, but most of them are anecdotal cases with varying outcomes. Atlantoaxial instability may result in a serious outcome, either acutely or chronically. Great attention should be paid to this condition. Surgical intervention of SD is one of the most challenging areas in orthopedic field, and will evolve with advance of orthopedic technology.
